# Effects of Modified Zhisou Powder on Airway Mucus Production in Chronic Obstructive Pulmonary Disease Model Rats with Cold-Dryness Syndrome

**DOI:** 10.1155/2018/7297141

**Published:** 2018-07-18

**Authors:** Gao Zhen, Wang Jing, Jing Jing, Dan Xu, Li Zheng, Fengsen Li

**Affiliations:** National Clinical Research Base of Traditional Chinese Medicine, Traditional Chinese Medicine Hospital Affiliated to Xinjiang Medical University, Urumqi 830000, China

## Abstract

*Objective. *In China, the Chinese medicine formula modified zhisou powder (MZP) is commonly used to treat COPD with cold-dryness syndrome (CDSCOPD) to relieve cough and sputum. However, the underlying mechanisms of MZP on treating CDSCOPD remain to be elucidated.* Methods*. COPD and CDSCOPD rat models were established; MZP was given to CDSCOPD rats in the last 7 days of the 97-day model establishment. Then the rats were subjected to lung function measurement. Pathological changes in lungs were observed through paraffin section and H&E staining. The mRNA and protein levels of AQP1, 4, and 5 and Muc5AC and Muc5B in lung were determined by quantitative RT-PCR and western blotting. NE levels was determined by ELISA.* Results*. The impaired lung functions were observed in rats exposed to cigarette smoke. Among all parameters evaluating lung functions, only tidal volume demonstrates a further decrease in CDSCOPD when compared with COPD, indicating further impaired pulmonary ventilation functions upon cold-dryness stimulation. The intervention of MZP effectively improved lung functions parameters, prevented the inflammations, and eliminated the increases of AQP4 and 5 and the decrease of Muc5AC in lung.* Conclusion.* MZP probably improves pulmonary functions in CDSCOPD through inhibiting lung inflammation, increasing expressions of AQPs, and decreasing Muc5AC expression in lung.

## 1. Background

Chronic obstructive pulmonary disease (COPD) is a disorder characterized with progressive airflow limitation caused by chronic inflammation in airways and lung parenchyma and is generally associated with symptoms such as cough, sputum production, and dyspnea [1]. Chronic mucus hypersecretion is not only related to respiratory tract infection that serves as one of the major risks of subsequent hospitalization but also associated with consistent decreases of FEV1 values in COPD patients [2, 3]. Furthermore, chronic cough, sputum production, and decreased FEV1 were proven to be independently associated with an increased risk of frequent exacerbations and hospitalization [4]. Therefore, airway mucus hypersecretion is not only a clinical symptom but also an independent risk factor affecting disease progression and prognosis prediction in lung diseases including COPD [5]. In chronic bronchitis and COPD, treatment with mucolytics led to reductions in acute exacerbations and days of illness [6]. In recent years, more and more attention has been paid to the effects of controlling airway mucus hypersecretion on the pathogenesis of COPD.

Traditional Chinese medicine remains an underexplored, yet potentially fruitful basis for COPD. Modified zhisou powder (MZP) is a famous traditional Chinese prescription that was originally recorded in a traditional Chinese Medicine Classic* Yixue Xinwu *of Qing Dynasty which was compiled in 1732. MZP is composed of 11 different herbs:* Nepeta cataria* L., 10g, Pericarpium Citri Reticulatae, 10g, Radix Glycyrrhizae Preparata, 10 g,* Platycodon grandiflorus*, 10g,* Stemona japonica*, 30g,* Aster tataricus* L. f., 20g,* Tussilago farfara* L., 20g,* Folium Perillae*, 10g,* Amygdalus communis* Vas, 15g,* Zingiber officinale* Roscoe, 10g, and* Fritillaria przewalskii* maxim ex Batal, 9g. It has been prescribed in treating “chronic cough.” In ancient China, the formula MZP has been used to treat lung diseases and relieve the symptoms like cough and sputum production, since it exhibits remarkably antitussive and expectorant effects [7]. In modern time, MZP was used to cure external wind-cold syndromes in patients with COPD or upper respiratory tract infection [8, 9]. Clinical observations and meta-analysis showed that MZP may be effective in treating postinfections cough [10, 11]. Animal study also showed that MZP improved the COPD associated cold-dryness syndromes, manifested by weakened sputum sound, increased PEF (peak expiratory flow) and EF50 (50% tidal volume expiratory flow) values, decreased inspiratory time (Ti) and expiratory time (Te), and delayed lung function decline [12]. And the antiasthmatic mechanisms were related to modified zhisou powder's significant reduction in contents of ET-1, NO, and EOS and the possible damage of lung tissue [13]. The cough and phlegm relieving functions of MZP might be attributed to the two major herb components in this formula,* Aster tataricus* L. f. and* Stemona sessilifolia* [14]. The root of* A. tataricus* has significant expectorant, antitussive, and anti-inflammatory effects [15]. And alkaloids extracted from root of* Stemona sessilifolia* have antitussive activity [16]. From the viewpoint of TCM, the cold-dryness syndromes are supposed due to the aberrant productions of body fluids like mucus in tissues. The cold-dryness syndrome affects the expressions of mucus-associated proteins like aquaporins and increases the secretion of mucins [12]. MZP improved the syndromes in lung by regulating body fluids metabolism in airways, which is called “moistening lung” in TCM [7, 17]. Therefore, MZP has been commonly used in treating COPD with cold-dryness syndrome, which is one of the most common COPD symptoms in northwest China according to the TCM methods of syndrome differentiation and classification in COPD [18].

In this study, a rat COPD model with cold-dryness syndrome was established. And the underlying mechanisms of effects of MZP on cold-dryness stimulated COPD were explored. Improved lung functions were found in COPD rats that received MZP intervention, accompanied with the expressions of increased aquaporin proteins and decreased mucin protein in lung tissues.

## 2. Materials and Methods

### 2.1. Drugs

The formula MZP was originally derived from the TCM book* Yixue Xinwu, *which was compiled in 1732 [19]. It was composed of 11 different herbs:* Nepeta cataria *L., 10 g, Pericarpium Citri Reticulatae, 10 g, Radix Glycyrrhizae Preparata, 10 g,* Platycodon grandiflorus*, 10 g,* Stemona japonica, *30 g,* Aster tataricus *L. f., 20 g,* Tussilago farfara *L., 20 g,* Folium Perillae*, 10 g,* Amygdalus communis Vas, *15 g,* Zingiber officinale *Roscoe, 10 g, and* Fritillaria przewalskii maxim ex Batal*, 9 g. The free decoction particles of herbs were purchased from Traditional Chinese Medicine Hospital Affiliated to Xinjiang Medical University.

### 2.2. Animals

Eighty male Wistar rats (150±20 g) were supplied by the Center of Experimental Animals, Xinjiang Medical University, Urumqi, China. Rats were acclimatized for 3 days to the room temperature (25 ± 3°C) and relative humidity of 60.0%-80.0%. Then the rats were randomly divided into five groups (control, normal control group (n = 15); COPD, COPD model group (n = 20); CDSCOPD, group of COPD with cold-dryness syndrome (n = 15); MZPCOPD, group of COPD with cold-dryness syndrome receiving MZP intervention (n = 15); and SR, group of COPD with cold-dryness syndrome receiving spontaneous recovery (n = 15). All experimental procedures were approved by the Animal Care and Use Committee of Xinjiang Medical University.

### 2.3. Rat Model Preparation

The COPD rat model was prepared according to the method described previously [12]. The rats in groups COPD, CDSCOPD, MZPCOPD, and SR were exposed to cigarette smoke (commercial Hatamen brand filtered cigarettes (China Tobacco Shandong Industrial Co. Ltd., Qingdao, China; each cigarette yields 11 mg tar, 0.8 mg nicotine, and 13 mg CO) in a 420 L poisoning cabinet connected to a smoking apparatus, which supplied smoke from one cigarette at 15 sucks/min. The cigarette smoke exposure was given 1 h each time, twice per day, in days 1 to 29 and days 31 to 97 (for SR rats, it was in days 1-29 and 31-90.) with intratracheal drip of elastase (20 U in 0.8 ml saline per 100 g body weight; Shanghai Aladdin Biochemical Technologies Inc.) on day 30. The rats in groups CDSCOPD, MZPCOPD, and SR were placed in an artificial climate test chamber (FLI-2000H artificial climate test chamber (EYELA, Japan)) with stable temperature (6±1°C) and relative humidity (25.0%-32.8%) at each night (10 h) to induce cold-dryness syndrome.

### 2.4. Interventions

For MZP intervention, the rats in MZPCOPD group were given MZP from day 91 to day 97. The dose was calculated according to the “equivalent dose table for human and animal translation by body surface area” [20]. Thereby, the daily dose for a rat was 0.96 g per 200 g body weight. A total volume of MZP solution (5 ml in saline) was administrated through gavage twice a day, once in the morning and once in the evening. The rats in other groups were given an equivalent volume of saline simultaneously.

### 2.5. Pulmonary Function Measurement

The pulmonary functions were assessed using noninvasive pulmonary functionality test system (BUXCO MA1320 respiratory function test table (Buxco, Wilmington, North Carolina, USA) to measure the minute ventilation (MV), peak of inspiratory flow (PIF), peak of expiratory flow (PEF), ratio of expiratory/inspiratory time (Te/Ti), tidal volume (TV), enhanced pause (Penh), pause (PAU), and 50% tidal volume expiratory flow (EF50).

### 2.6. Pathological Examinations of Lung Tissues

The rats were sacrificed at day 97 after lung functions assessment. The same lobe of right lung from each rat was fixed in 10% formalin, routinely dehydrated, embedded in paraffin, and sectioned (2 *μ*m). Then the sections were subjected to H&E staining for histological morphology observation under microscope (Leica, Wetzlar, Germany).

### 2.7. Real-Time RT-PCR

Total RNA was extracted from lung tissues with TRIzol reagent (Invitrogen). Reverse-transcription was performed with 1000 ng total RNA by using Takara cDNA synthesis kit (Dalian, China). The relative mRNA levels of genes* AQP1, 4, *and* 5 *were determined with real-time quantitative PCR by using QuantiTect SYBR Green Kit (Qiagen) and primers listed in Table 1 and calculated using 2-^△△Ct^ method.

### 2.8. Western Blotting

The total proteins of rat lung tissues were extracted with RIPA lysis buffer. Equal amounts of total proteins (20 *μ*g) were separated in 12% SDS-PAGE, transferred onto PEDF membrane, and blotted with primary antibodies against AQP1 (sc-9878), AQP4 (sc-20812), AQP5 (sc-28628), Muc5AC (sc-71620), Muc5B (sc-135508), and *β*-actin (sc-8432) (all antibodies were from Santa Cruz Biotechnology). After incubation with corresponding secondary antibodies, the bands were visualized by using ECL, photographed, and subjected to density quantitation with Bio-Rad software. The relative expression levels of target proteins were estimated by normalizing against the density of *β*-actin (as internal control).

### 2.9. ELISA

A transverse incision of the inferior trachea was made and the right main bronchus ligated. This was used to inject 3 mL of physiological saline solution into the left lung. Perfusate was retrieved immediately after each perfusion (60%-70%) and filtered using a sterile bandage. Filtration was repeated three times to collect BALF for centrifugation, frozen at -70°C. ELISA was used to detect NE, following the kit instructions exactly.

### 2.10. Statistical Analysis

The statistical analysis was performed by using SPSS software for Windows (IBM SPSS version 20.0). Due to the wide variability of the respiratory function and as a preparation for statistical analysis, the median of each 30-second period was calculated for each animal. The use of median values permitted us to discard the influence of occasional extreme values. The data were subjected to one-way ANOVA analysis if they were normally distributed and expressed as mean ±* SD*. Significant differences among groups were evaluated by using the least significant difference (LSD) range test or Dunnett T3 test. Otherwise, the data were analyzed with Wilcoxon signed-rank test and expressed as median (IQR). P<0.05 and P<0.01 were considered as significant and extremely significant difference, respectively.

## 3. Results

### 3.1. MZP Prevented Lung Functions Decline in COPD Rats with Cold-Dryness Syndrome

After the COPD rat models were established, the rats were subjected to noninvasive pulmonary function assessments. As shown in Tables 2 and 3, compared with normal rats, cigarette smoke exposure induced COPD-like symptoms including the decreases of MV, PIF, PEF, and TV and the increases of Penh and PAU, though no obvious difference was found between rats from COPD and CDSCOPD groups. The parameter TV showed more decrease in CDSCOPD than in COPD rats. However, the parameters EF50 and the ratio of Te/Ti, both reflecting the air flow limitation in expiratory, were not affected in COPD rats, though declining trend of EF50 was found in COPD and CDSCOPD rats. MZP improved the lung functions by eliminating the decreases of MV, PIF, PEF, and TV and the increases of Penh and PAU. However, in the parameters Penh and PAU, when comparing MZPCOPD with SR rats, the improved effects of withdrawal of cigarette smoke exposure were better than MZP intervention under simultaneous cigarette smoke exposure. But, in other parameters, the effects of spontaneous recovery showed no difference from MZP intervention, except for MV, for which MZP intervention showed better effects than spontaneous recovery.

### 3.2. MZP Alleviated Lung Inflammation in COPD Rats

**Figure 1:**
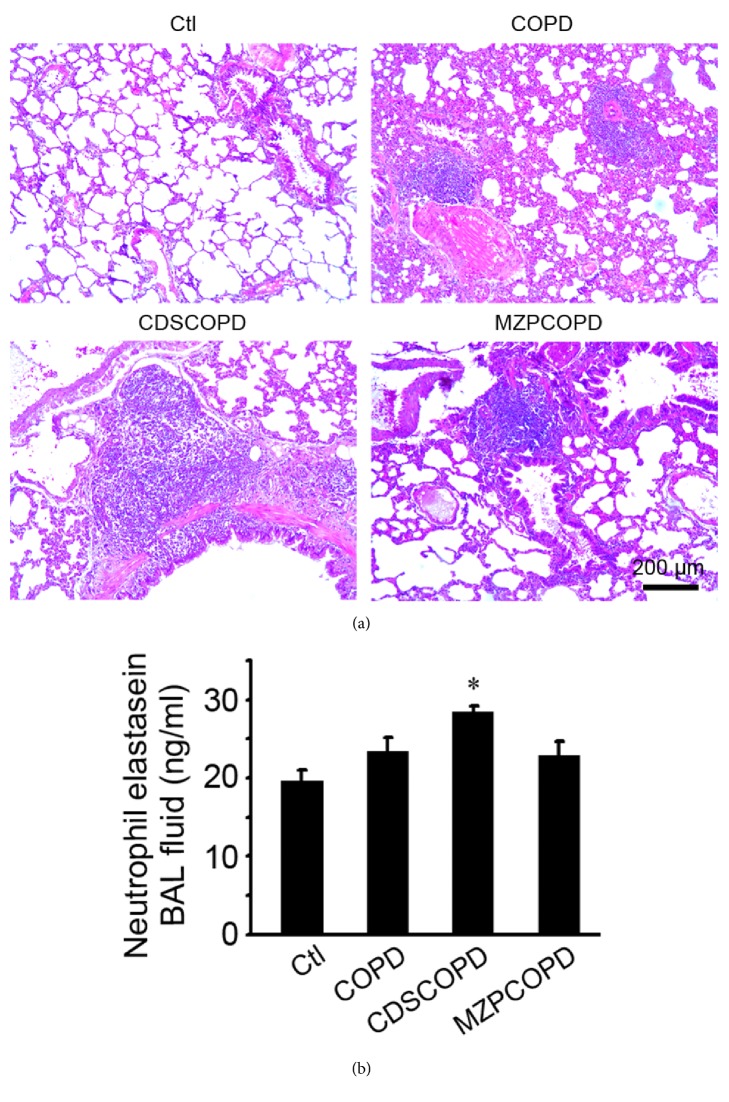
**Inflammations in lung.** (a) Paraffin lung sections from groups (Ctl, COPD, CDSCOPD, and MZPCOPD) were stained with hematoxylin-eosin and photographed under a microscope (Leica, Wetzlar, Germany). The representative photos were from one of six to ten rats in each group. (b) The neutrophil elastase levels in BAL fluid. Data was expressed as mean ± SD (n=3). ^*∗*^P<0.01 for CDSCOPD versus the other three groups.

In order to evaluate the effects of modified zhisou powder on lung inflammation induced by cigarette smoke exposure plus cold-dryness stimulation, the lung sections from four groups, Ctl, COPD, CDSCOPD, and MZPCOPD, were subjected to H&E staining. In normal rats' lung, the pulmonary alveoli were in normal size with appropriate wall thickness and very few infiltrated inflammatory cells were found in peribronchiolar area. Obvious inflammatory cell infiltration and granuloma formation in airway could be seen in lungs of COPD rats. The inflammatory responses in lung became more severe in CDSCOPD rats. When compared with COPD and CDSCOPD rats, obviously fewer inflammatory cells infiltration was seen in lung from MZPCOPD rats (Figure 1(a)). The level changes of neutrophil elastase in BAL fluids were consistent with the differential lung inflammation in each group (Figure 1(b)).

### 3.3. MZP Prevented AQPs Downregulations and Mucins Upregulations upon Cigarette Smoke Exposure

**Figure 2:**
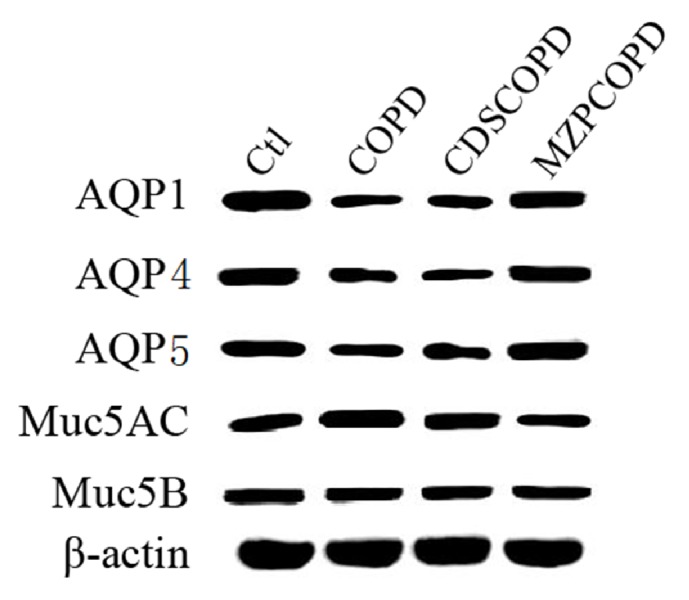
The changes of AQPs and mucins in lung tissues from COPD rats. Comparison of AQP1, AQP4, AQP5, Muc5AC, and Muc5B protein expression in lung tissue among groups. Note: COPD: chronic obstructive pulmonary disease, CDSCOPD: COPD with cold-dryness syndrome, and MZPCOPD: CDSCOPD treated by modified zhisou powder.

The mRNA or protein levels of AQP1, 4, and 5 and Muc5AC and Muc5B in lung tissues were determined by quantitative RT-PCR or western blotting, respectively. As shown in Tables 4 and 5, cigarette smoke exposure downregulated the mRNA and protein levels of AQPs in lung. The cold-dryness stimulation did not further decrease the expressions of AQPs when comparing CDSCOPD with COPD groups. The administrations of modified zhisou powder partially prevented the loss of AQP4 and 5 proteins in CDSCOPD rats' lung (Figure 2).

For the proteins Muc5AC and Muc5B, cigarette smoke exposure induced obvious productions of Muc5AC but not Muc5B in lung. However, similar to the situations in AQPs, no obvious difference on Muc5AC level changes was observed between COPD and CDSCOPD rats. Modified zhisou powder demonstrated potential (a trend towards preventing Muc5AC upregulation) but not obvious effects on eliminating Muc5AC upregulation in CDSCOPD rats' lung (Table 5, P=0.19 for CDSCOPD versus MZPCOPD).

## 4. Discussion

In TCM clinical practice, the geographical environment has been considered as one of major factors impacting syndrome differentiation and clinical strategy decision of certain disease [21]. COPD with cold-dryness syndrome is the most common COPD syndrome seen in northwest China, where the climate is cold and rainless [18]. In this study, we established COPD and COPD with cold-dryness syndrome model rats and proved that Chinese medicine formula MZP is effective in elevating lung functions in rats of COPD with cold-dryness syndrome, possibly through eliminating lung inflammatory responses and preventing the loss of AQP4/5 and the hyperproduction of Muc5AC in lung. Low environmental temperature was supposed to elevate the risk of chronic bronchopneumonia through attenuating the airway defense capability and enhancing the airway hyperresponsiveness, which might contribute to the COPD development [22]. However, though the differences on behaviors could be observed between COPD and CDSCOPD rats, the latter needed to drink more water daily [12]; for all parameters estimating lung functions in this study, only tidal volume in CDSCOPD rats was less than that in COPD rats, indicating further that impaired lung gas exchange occurred in CDSCOPD rats. For the parameters MV, PIF, PEF, Penh, and PAU, although significant decreases or increases occurred in both COPD and CDSCOPD rats when compared with normal rats, no difference was observed between COPD and CDSCOPD rats. We supposed that the reason might be attributed to the mild COPD symptoms in this study, which were due to the low amount of cigarettes used to produce cigarette smoke (only two pieces per day) when compared with previous study [23] or due to the rats as animals relatively resistant to development of COPD [24]. Therefore, intratracheal drip of elastase, an enzyme inducing emphysema phenotype, was used to promote the model establishment [25].

In COPD with cold-dryness syndrome, it was supposed that cold temperature and low humidity play pivotal roles in promoting the onset of respiratory tract infections. Research found that COPD rats may be more susceptible to cold stress. Cold stress may aggravate PM2.5-induced toxic effects in the lung of COPD rats through increasing Ang-II/NF-*κ*B signaling pathway and suppressing Nrf2 signaling pathway [26]. Cold and dry air contributed to the excessive productions of mucus and drying of mucus in airway, which led to the impaired mucociliary clearance of airway surface, a critical innate defense mechanism in upper airway, and ultimately the occurrence of airway obstruction [27–29]. The proteins AQPs not only control the volume of liquid secreted to the airway cavity but also impact the expressions of mucin [30]. Similar to previous study, we also found decreases of AQPs and increases of mucin protein in COPD rats' lung [31]. However, although no difference was found between COPD and CDSCOPD rats, we cannot exclude the possibility that longer time of cold-dryness stimulation might impact expressions of AQPs and mucins in lung.

MZP demonstrated protective effects on COPD, manifested by improved lung functions and eliminated lung inflammations. In this study, we did not evaluate the effects of MZP on COPD without cold-dryness syndrome. However, the results at least demonstrated that MZP has protective effects on development of COPD with cold-dryness syndrome, which could be attributed to its regulating effects on AQPs and, probably, Muc5AC. Among the MZP components, alkaloids separated from* Stemona japonica *have insecticidal and antitussive activities [32, 33]. Similar effects could be found in another major component of this formula,* Aster tataricus *L. f., from which root antitussive and anti-inflammatory mixture could be extracted [15]. Anti-inflammatory, antiviral, antiapoptotic, and/or antioxidant activities could also be found in minor components like* Nepeta cataria *L. [34], Pericarpium Citri Reticulatae [35, 36], Radix Glycyrrhizae Preparata [37],* Tussilago farfara *L. [38],* Folium Perillae* [39], and* Zingiber officinale* Roscoe [40]. Furthermore, saponin derived from root of* Platycodon grandiflorus* and alkaloids extracted from* Fritillaria* plants inhibit Muc5AC expression [41]. However, more works are needed to identify the components regulating AQPs expression, because this effect has not been attributed to any of the herbs or herbs extracts involved in this formula.

As a result of aquaporins upregulation and Muc5AC downregulation, it is reasonable to propose that, except for anti-inflammatory effects, MZP controls the risk of COPD by inhibiting airway mucus obstruction, which could induce chronic cough and expectoration, while a paroxysmal cough is an independent risk factor for COPD [42, 43]. Notably, from the improved parameters like MV, PEF, PIF, TV, and EF50 in MZPCOPD rats, we can say that the pulmonary ventilation functions were elevated upon MZP intervention.

## 5. Conclusions

The TCM formula MZP improves the pulmonary functions probably through inhibiting lung inflammation, increasing expressions of AQPs, and decreasing Muc5AC expression in lung of COPD model rats with cold-dryness syndrome.

## Figures and Tables

**Table 1:** 

Gene		Sequence (5'→3')	Product size (bp)
AQP1	Forward	GACTACACTGGCTGTGGGATCAA	115
	Reverse	CCAGGGCACTCCCAATGAA	

AQP4	Forward	AGGCAATGTGTGCACTGCTCTA	120
	Reverse	AAGGTGTCAACGTCACACAACAA	

AQP5	Forward	CATGGTGGTGGAGTTAATCTTGA	161
	Reverse	CATGGAACAGCCGGTGAAGTAG	

*β*-Actin	Forward	GGAGATTACTGCCCTGGCTCCTA	150
	Reverse	GACTCATCGTACTCCTGCTTGCTG	

**Table 2:** The parameters indicating lung functions (part 1).

Group	N	MV (ml)	PIF (ml/s)	PEF (ml/s)	TV (ml)	EF50 (ml/s)	Penh
Ctl	6	163.34 (118.6-485.73)	11.62 (8.28-37.10)	6.92 (4.85-24.60)	1.31 (0.81-2.7)	0.5 (0.25-1.34)	0.45 (0.34-0.56)
COPD	10	133.57 (110.42-165.36)^*∗∗*^	10.14 (8.17-12.31)^*∗*^	6.28 (5.31-7.50)^*∗*^	1.18 (1.01-1.37)^▲▲^	0.37 (0.27-0.52)	0.54 (0.43-0.64)^*∗∗*^
CDSCOPD	10	134.45 (106.23-184.78)^*∗∗*^	10.22 (8.89-12.13)^*∗*^	6.49 (5.50-7.72)	1.03 (0.87-1.19)^*∗∗◆◆*^	0.42 (0.28-0.56)	0.58 (0.42-0.75)^*∗∗*^
MZPCOPD	10	186.78 (146.69-284.24)^▲▲◆◆*◇*^	12.78 (10.44-19.26)^▲▲◆◆^	8.2 (6.31-12.01)^▲▲◆◆^	1.5 (1.14-2.05)^▲▲◆◆^	0.51 (0.35-0.91)^▲▲◆◆^	0.45 (0.35-0.59)^▲▲◆◆*◇◇*^
SR	10	171.51 (124.71-232.68)^▲▲◆◆^	11.75 (9.12-16.58)^▲▲◆◆^	8.48 (6.24-10.67)^▲▲◆◆^	1.35 (1.17-1.89)^▲▲◆◆^	0.52 (0.36-0.69)^▲◆◆^	0.62 (0.47-0.71)^*∗∗◆*^

The parameters indicating pulmonary functions were assessed using noninvasive pulmonary functionality test system. Data were expressed as median (IQR) and analyzed with Wilcoxon signed-rank test. ^*∗*^P < 0.05 and ^*∗∗*^P < 0.01, when compared with control group; ^▲^P < 0.05 and ^▲▲^P < 0.01, when compared with CDSCOPD group; ^◆^P < 0.05 and ^◆◆^P < 0.01, when compared with COPD group; ^*◇*^P < 0.05 and ^*◇◇*^P < 0.01, when compared with the spontaneous recovery group.

**Table 3:** The parameters indicating lung functions (part 2).

Group	N	Te/Ti	PAU
Ctl	6	1.82±0.39	0.68±0.16
COPD	10	1.86±0.51	0.78±0.15^*∗∗*^
CDSCOPD	10	1.84±0.52	0.80±0.18^*∗∗*^
MZPCOPD	10	1.74±0.47	0.70±0.17^▲▲◆◆*◇◇*^
SR	10	1.70±0.41	0.80±0.14^*∗∗*^

The parameters indicating pulmonary functions were assessed using noninvasive pulmonary functionality test system. Data were expressed as mean ± SD and analyzed with one-way ANOVA and LSD tests. ^*∗*^P < 0.05 and ^*∗∗*^P < 0.01, when compared with control group; ^▲^P < 0.05 and ^▲▲^P < 0.01, when compared with CDSCOPD group; ^◆^P < 0.05 and ^◆◆^P < 0.01, when compared with COPD group; ^*◇*^P < 0.05 and ^*◇◇*^P < 0.01, when compared with the spontaneous recovery group.

**Table 4:** The changes of AQPs mRNA in lung tissues from COPD rats.

Group	*N*	AQP1 mRNA	AQP4 mRNA	AQP5 mRNA
COPD	7	0.23±0.10^*∗∗*^	0.62±0.31	0.20±0.08^*∗∗*^
CDSCOPD	7	0.27±0.14^*∗∗*^	0.46±0.23^*∗∗*^	0.20±0.11^*∗∗*^
MZPCOPD	7	0.42±0.16^*∗∗*^	0.46±0.22^*∗∗*^	0.50±0.22^*∗∗*^
Control	7	1.00±0.00	1.00±0.00	1.00±0.00
*F*		65.25	9.08	60.24

Notes: The relative mRNA levels of AQP1, AQP4, and AQP5 in lung tissues were determined with quantitative RT-PCR with *β*-actin as internal control. Data was expressed as mean ± SD and analyzed with one-way ANOVA and LSD tests or Dunnett T3 test. ^*∗∗*^P < 0.01 versus control group.

**Table 5:** The changes of AQPs and mucins in lung tissues from COPD rats.

Group	*N*	AQP1	AQP4	AQP5	MUC5AC	MUC5B
COPD	5	0.24±0.10	0.24±0.12	0.20±0.07	3.20±0.59^##^	1.29±0.15
CDSCOPD	5	0.28±0.13	0.27±0.17	0.16±0.09	3.08±0.86^#^	1.25±0.54
MZPCOPD	5	0.42±0.12	0.64±0.17^*∗*^	0.54±0.13^*∗*^	1.89±0.49	1.25±0.29
Control	5	1.07±0.15^*∗∗*^	0.97±0.19^*∗∗*^	1.00±0.19^*∗∗*^	0.99±0.19	1.08±0.23
F		46.74	22.07	46.78	16.11	0.41

Notes: The protein levels of AQP1, AQP4, AQP5, Muc5AC, and Muc5B in lung tissues were determined by using western blotting and the bands were subjected to density quantitation. The relative protein levels to *β*-actin were calculated and expressed in arbitrary units. The results were from representative rat randomly selected from each group (n=5 rats per group). For band relative protein level calculation, data was expressed as mean ± SD and analyzed with one-way ANOVA and LSD tests or Dunnett T3 test. ^*∗∗*  ^P < 0.01 for Ctl versus other three groups in AQP1, AQP4, and AQP5; ^*∗*^P < 0.05 for CDSCOPD versus MZPCOPD in AQP4 and AQP5; ^##^P < 0.01 and ^#^P < 0.05 for protein Muc5AC when compared with Ctl group.

## Data Availability

The data used to support the findings of this study are included within the article.

## Abbreviations

COPD: Chronic obstructive pulmonary disease
CDSCOPD: COPD group with cold-dryness syndrome
MZPCOPD: COPD group with MZP intervention
SR: Spontaneous recovery group
AQP: Aquaporin
MUC: Mucin
TV: Tidal volume
Penh: Enhanced pause
PAU: Pause
EF50: 50% tidal volume expiratory flow
PEF: Peak expiratory flow
MV: Minute ventilation.
